# Anemia and opportunistic infections in hospitalized people living with HIV: a retrospective study

**DOI:** 10.1186/s12879-022-07910-5

**Published:** 2022-12-07

**Authors:** Bo Xie, Wei Huang, Yanling Hu, Yanyun Dou, Luman Xie, Yong Zhang, Shanfang Qin, Ke Lan, Xianwu Pang, Hong Qiu, Lanxiang Li, Xihua Wei, Zengjing Liu, Zhihao Meng, Jiannan Lv

**Affiliations:** 1 School of Information and Management, Guangxi Medical University, Nanning, 530021 Guangxi China; 2 Guangxi Clinical Center for AIDS Prevention and Treatment, Chest Hospital of Guangxi Zhuang Autonomous Region, No. 8 Yangjiaoshan Road, Liuzhou, 545005 Guangxi China; 3 Institute of Life Sciences, Guangxi Medical University, Nanning, 530021 Guangxi China; 4 Center for Genomic and Personalized Medicine, Guangxi Key Laboratory for Genomic and Personalized Medicine, Guangxi Collaborative Innovation Center for Genomic and Personalized Medicine, Guangxi Medical University, Nanning, 530021 Guangxi China; 5 Basic Medical College of Guangxi Medical University, Nanning, 530021 Guangxi China

**Keywords:** HIV, Anemia, Opportunistic infection, Tuberculosis, Penicillium marneffei

## Abstract

**Background:**

There is a high prevalence of anemia among people living with HIV in Guangxi, China. Therefore, we investigated anemia and opportunistic infections in hospitalized people living with HIV and explored the risk factors related to anemia in people living with HIV to actively prevent anemia in people living with HIV.

**Methods:**

We retrospectively studied people living with HIV admitted to Guangxi Chest Hospital from June 2016 to October 2021. Detailed information on the sociodemographic and clinical features of the participants was collected. The X^2^ test was used to compare the prevalence between the anemic and non-anemic groups. The logistic regression analysis was applied to exclude confounding factors and identify factors related to anemia.

**Results:**

Among 5645 patients with HIV, 1525 (27.02%) had anemia. The overall prevalence of mild, moderate, and severe anemia was 4.66%, 14.08%, and 8.27%, respectively. The factors significantly related to increased risk of anemia were CD4 count < 50 cells/µl (aOR = 2.221, 95% CI = [1.775, 2.779]), CD4 count 50–199 cells/µl (aOR = 1.659, 95% CI = [1.327, 2. 073]), female (aOR = 1.644, 95% CI = [1.436, 1.881]) co-infected with HCV (aOR = 1.465, 95% CI = [1.071, 2.002]), PM (aOR = 2.356, 95% CI = [1.950, 2.849]), or TB (aOR = 1.198, 95% CI = [1.053, 1.365]).

**Conclusions:**

Within Guangxi of China, 27.02% of hospitalized people living with HIV presented with anemia. Most patients with anemia were in the mild to moderate stage. The low CD4 count, female gender, and concomitant infection with Penicillium marneffei, Hepatitis C virus, or Tuberculosis were independent correlates of anemia. Thus, these findings would be helpful to clinicians in preventing and intervening in anemia in people living with HIV.

## Background

Hematological abnormalities are a common complication of HIV infection [1]. It becomes increasingly frequent as the disease progresses, and anemia is one of the most common blood abnormalities in HIV-infected patients [2]. In addition, anemia could negatively affect the life quality and mortality of patients [3]. Therefore, taking effective measures to improve anemia can aid the survival and prognosis of HIV-infected patients [4–6].

The currently reported associated risk elements for anemia include gender, age, income level, impact of antiretroviral therapy (ART) drugs, CD4 count, HIV viral load, and opportunistic infections (OIs) [7, 8]. Moreover, anemia may cause a range of clinical symptoms, including fatigue, weakness, dizziness, and drowsiness, which greatly affect the quality of life of patients [3, 9]. Anemia could also lead to impaired physical function and psychological distress [10]. However, it is difficult to treat anemia in HIV-infected patients because of the complexity and variety of causes.

The prevalence of anemia among people living with HIV in China could range from 9.8% to 55% [11, 12]. However, mild anemia is often not paid enough attention by clinical workers. In a study of adults with newly infected HIV in China, the total prevalence of anemia was 51.9% [13]. Thus, this study reported that anemia was related to an ethnic minority, age, and CD4 count. Meanwhile, a study from Beijing, China, which investigated people living with HIV not receiving ART, reported that the total prevalence of anemia in people living with HIV was only 9.8% [11]. This study discovered that independently correlated elements with anemia included females, elder age, depressed body mass index (BMI), and higher viral load. Moreover, an epidemiologic survey on anemia in people living with HIV in Xinjiang [14], China, indicated that 38.9% of HIV-infected patients suffered from anemia at the beginning of the ART. It revealed that Uyghurs, females, depressed CD4 count, decreased BMI, a previous history of tuberculosis (TB), and oral candidiasis were independently correlated risk factors for anemia.

Although the profile and risk elements of anemia in people living with HIV have been one of the popular issues of research, few studies have focused on the relationship between OIs (particularly Penicillium marneffei (PM), TB, hepatitis B virus (HBV), and hepatitis C virus (HCV)) and anemia in people living with HIV. OIs could increase the probability of hospitalization and mortality in people living with HIV and could also affect their quality of treatment outcomes. However, the relationship between anemia and OIs in people living with HIV has not been recorded and reported in most parts of China. Consequently, identifying the relationship between anemia and OIs in people living with HIV is important to rationally manage people living with HIV. This study aimed to investigate the prevalence of anemia and its related factors among HIV inpatients in Guangxi Zhuang Autonomous Region (Guangxi) Chest Hospital, which is a designated treatment hospital for major infectious diseases in Guangxi, China.

## Methods

### Study design and patient selection

We conducted a retrospective study on hospitalized people living with HIV in Guangxi Chest Hospital between June 2016 and October 2021. This study included adult people living with HIV aged 18 years and older. Meanwhile, participants with insufficient information and pregnancy were excluded. All participants were diagnosed as HIV infection as verified by laboratory testing and the diagnosis met the national guidelines for HIV diagnosis.

### Data collection and definitions

In this study, recordings of blood tests from HIV-infected hospitalized patients were extracted from the electronic medical record system of Guangxi Chest Hospital. Females or males with hemoglobin values of < 110 g/L or < 120 g/L were identified as anemic, respectively. Anemia was further categorized into mild anemia (hemoglobin 90–109 g/L in females or 90–119 g/L in males), moderate anemia (hemoglobin 60–89 g/L), and severe anemia (hemoglobin < 60 g/L).

Meanwhile, the standards for TB diagnosis were consistent with the World Health Organization (WHO) guidelines for the control of TB [15]. TB was confirmed when mycobacterium tuberculosis could be detected in a biological sample. Hence, the clinical symptoms of TB contribute to the diagnosis. In addition, TB was often associated with systemic symptoms such as low-grade fever, night sweats, loss of appetite, wasting, and weakness. The diagnosis was further confirmed by diagnostic imaging (X-rays, CT scan, etc.) and laboratory tests. All included samples were clinically confirmed TB patients.

The diagnostic criteria for PM infection were the separation and confirmation of PM in a biological sample. Meanwhile, hepatitis B and C were clinically confirmed by the presence of antigenic antibodies in the blood and the detection of the virus. Cryptococcal infection was preliminarily filtered by testing for the presence of cryptococcal antigens in the blood. Moreover, the diagnosis of cryptococcosis was further confirmed by finding cryptococci under the microscope. Meanwhile, the criterion for diagnosis of cytomegalovirus infection was a positive cytomegalovirus antigen blood test.

### Statistical analysis

SPSS 24.0 (SPSS Inc., Chicago, USA) was used for further analysis of the data collected. Means ± SD was used to represent continuous variables. In addition, numbers (percentages) were used to denote categorical variables, and the percentage of participants with different levels of anemia was represented by a bar chart. Moreover, trends in the prevalence of OIs were represented with line graphs. The Pearson X^2^ test or X^2^ test or Fisher’s exact test was applied to compare the prevalence of different groups of anemia. Furthermore, a multifactorial logistic regression analysis was conducted to determine the correlation between the included variables and anemia. The ratio (OR) and 95% confidence intervals (CIs) determined the strength of the correlation between the included variables and anemia. Gender, age, CD4 count, HBV, HCV, PM, TB, Cryp, candidiasis, and CMV were the relevant factors included in the study. The statistical test was two-sided, and P < 0.05 was considered statistically significant.

## Result

### Patient characteristics

This study included a total of 5645 HIV-infected patients with ages ranging from 18 to 93 years and their average age was (54 ± 13.9) years. There were 4,321 males (76.4%) and 1324 females (23.6%). Most of the patients had their usual place of residence in the rural areas (75.52%) and more than half (51.41%) were ethnic minorities, with Zhuang (44.80%) constituting the majority. Approximately, 71.57% of the participants had CD4 counts of < 200 cells/µl. The essential features of the study participants were presented in Table 1.

**Table 1:** Socio-demographic characteristics of people living with HIV

Variables		Total Num (%)	No anemia (%)	Mild anemia (%)	Moderate anemia (%)	Severe anemia (%)	X^2^	P-value
Age, years							32.267	< 0.001
18–39		955 (16.92)	669 (70.05)	32 (3.35)	160 (16.75)	94 (9.85)		
40–59		2545 (45.08)	1861 (73.12)	107 (4.21)	342 (13.44)	235 (9.23)		
≥ 60		2145 (38.00)	1590 (74.13)	124 (5.78)	293 (13.66)	138 (6.43)		
Sex							35.558	< 0.001
Female		1324 (23.45)	891 (67.30)	69 (5.21)	211 (15.94)	153 (11.56)		
Male		4321 (76.55)	3229 (74.73)	194 (4.49)	584 (13.52)	314 (7.27)		
Ethnicity							5.982	0.425
Han		2743 (48.59)	2027 (73.90)	125 (4.56)	364 (13.26)	227 (8.28)		
Zhuang		2529 (44.80)	1812 (71.65)	124 (4.90)	383 (15.15)	210 (8.30)		
Other (minority)		373 (6.61)	281 (75.34)	14 (3.75)	48 (12.87)	30 (8.04)		
Marital status							29.429	< 0.001
Unmarried		1359 (24.07)	949 (69.83)	60 (4.42)	217 (15.97)	133 (9.78)		
Married		3237 (57.35)	2433 (75.16)	162 (5.00)	411 (12.70)	231 (7.14)		
Divorce and widowhood		1049 (18.58)	738 (70.35)	41 (3.91)	167 (15.92)	103 (9.82)		
Residence							6.605	0.086
Urban		1382 (24.48)	1034 (69.83)	56 (4.42)	171 (15.97)	121 (9.78)		
Rural		4263 (75.52)	3086 (75.16)	207 (5.00)	624 (12.70)	346 (7.14)		
CD4 counts, cells/μl							128.558	< 0.001
< 50		2258 (40.00)	1496 (66.25)	102 (4.52)	413 (18.29)	247 (10.94)		
50–199		1782 (31.57)	1322 (74.19)	94 (5.27)	242 (13.58)	124 (6.96)		
200–350		867 (15.36)	691 (79.70)	39 (4.50)	85 (9.80)	52 (6.00)		
> 350		738 (13.07)	611 (82.79)	28 (3.79)	55 (7.45)	44 (5.97)		

### Description of OIs among HIV-infected individuals

**Fig. 1:**
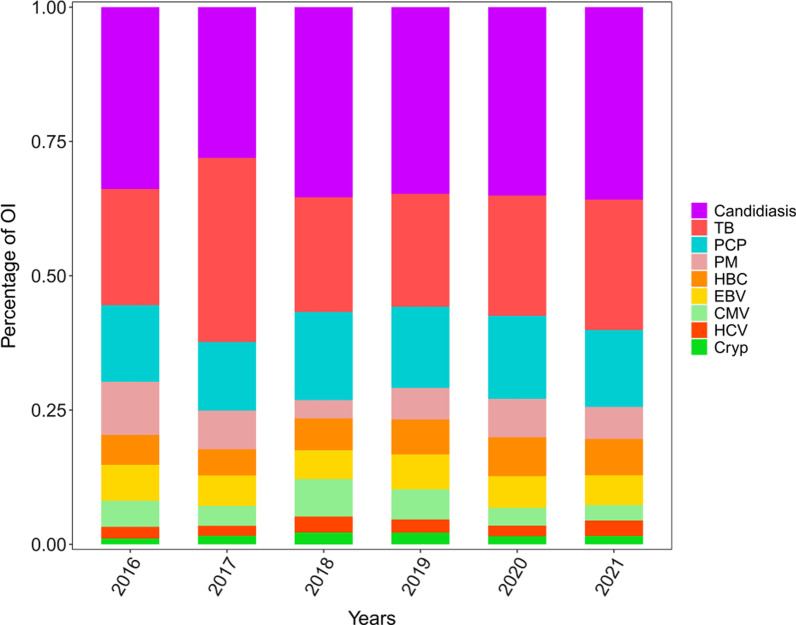
The prevalence of opportunistic infections in HIV-infected patients for each year from 2016 to 2021

**Fig. 2:**
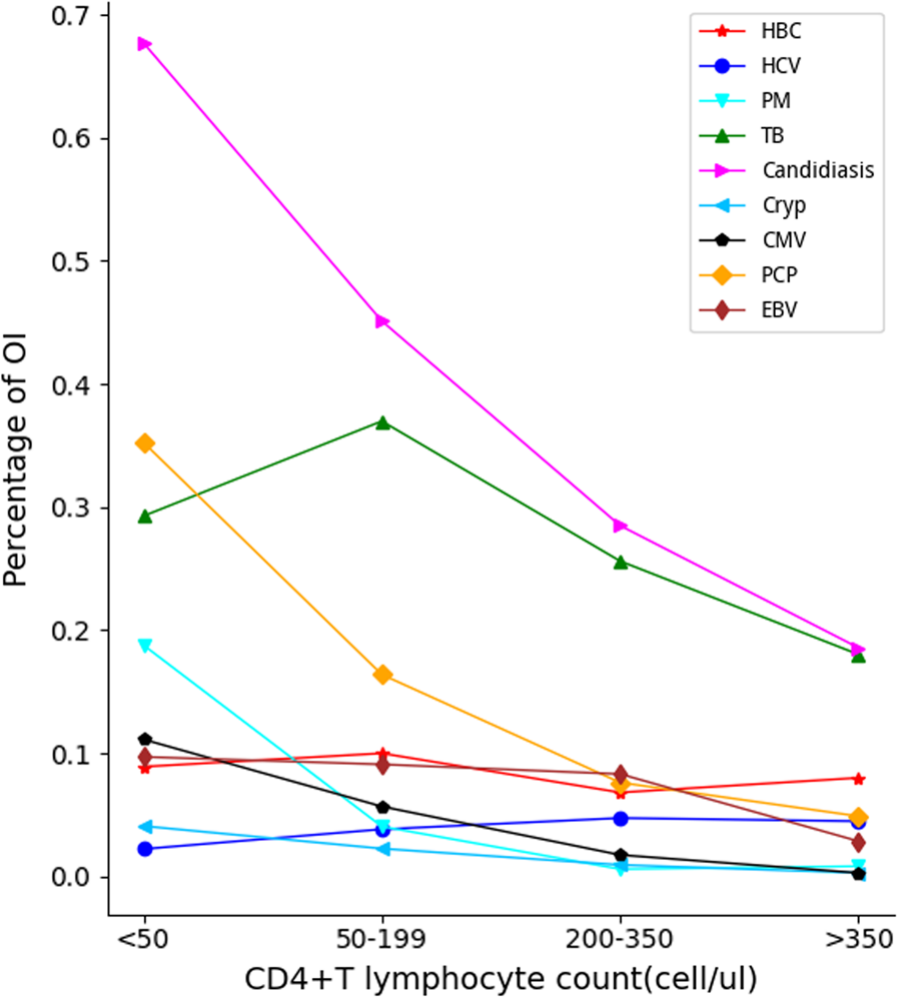
Prevalence of opportunistic infections at different CD4 count levels

The prevalence of OIs in HIV-infected patients annually from 2016 to 2021 was 79.63%, 80.30%, 75.74%, 75.89%, 68.67%, and 69.83%, respectively. The prevalence of OIs is decreasing year by year. OIs that accounted for most cases were candidiasis, TB, and pneumocystis pneumonia (PCP), separately (Fig. 1). The prevalence of OIs in participants with CD4 counts of > 350, 200–350, 50–199, and < 50 cells/µl was 90.92%, 75.36%, 54.79%, and 43.36%, respectively, thereby indicating a decreasing trend with increasing CD4 counts. The highest prevalence was in Candida infection (Fig. 2).

### Prevalence of anemia in HIV patients

**Fig. 3:**
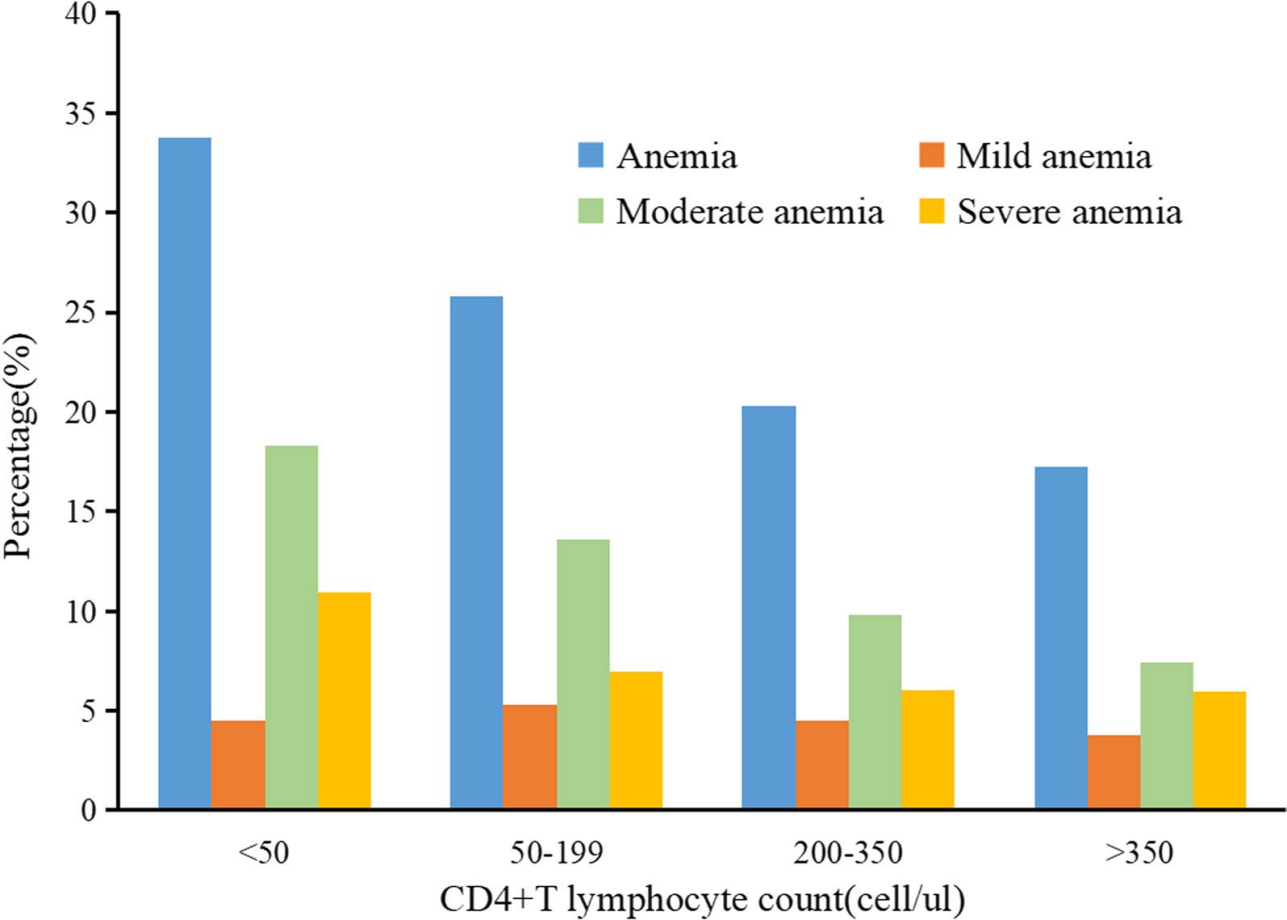
Prevalence of anemia at different CD4 count levels

Among 5645 HIV patients, 1525 (27.02%) had anemia. The total percentages of mild, moderate, and severe anemia were 4.66%, 14.08%, and 8.27%, respectively. Approximately, 25.27% and 32.70% of HIV-infected males and females were anemic, respectively (Table 1). The prevalence of anemia was 17.21%, 20.30%, 25.81%, and 33.75% in patients with CD4 counts of > 350, 200–350, 50–199, and < 50 cells/µl, respectively. Moderate and severe anemia prevalence gradually increased as CD4 count declined (Fig. 3).

**Fig. 4:**
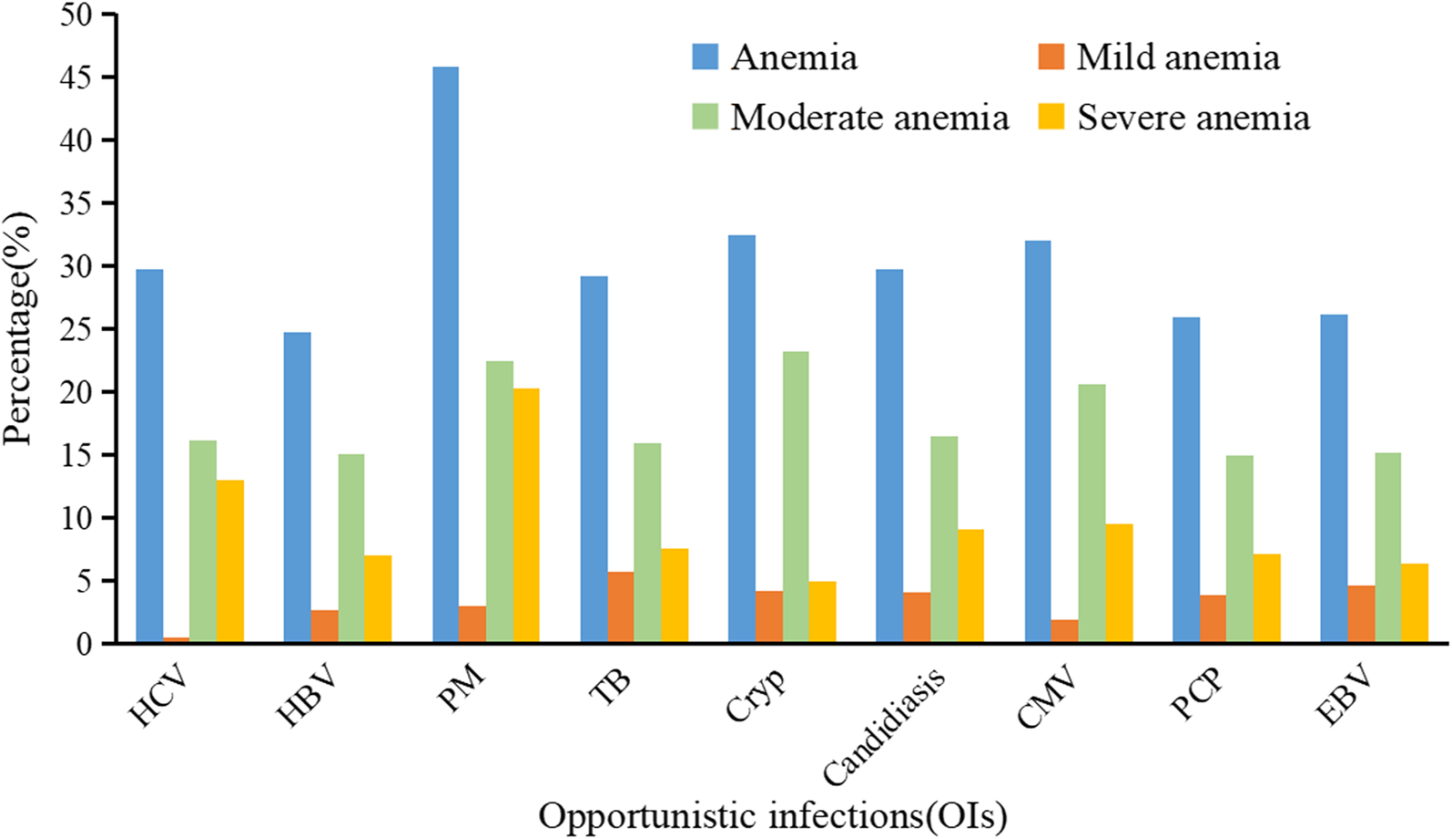
Prevalence of anemia in HIV patients with different OIs

HIV patients co-infected with TB (29.19%) have higher prevalence of anemia than in HIV-infected patients without TB (26.10%). Mild and moderate anemia was more commonly observed in patients with HIV co-infected with Cryp. A significant difference was found in the prevalence of anemia in HIV co-infected patients with PM (45.76%) compared to HIV co-infected patients without PM (25.17%). The prevalence of anemia in HIV patients with different OIs was shown in Fig. 4. Moreover, there were more frequent cases of moderate and severe anemia in HIV patients co-infected with PM. The prevalence of anemia was slightly higher in study subjects with HIV co-infected with HCV (29.69%) than in study subjects with HIV without HCV infection (26.92%) (Table 2).

**Table 2:** Univariate analysis of opportunistic infections associated with anemia

Variables	Total Num (%)	No anemia (%)	Mild anemia (%)	Moderate anemia (%)	Severe anemia (%)	X^2^	P-value
HCV						13.517	0.004
Positive	192 (2.39)	135 (70.31)	1 (0.52)	31 (16.15)	25 (13.02)		
Negative	5453 (70.59)	3985 (73.08)	262 (4.80)	764 (14.01)	442 (8.11)		
HBV						6.658	0.084
Positive	497 (8.80)	374 (75.25)	13 (2.62)	75 (15.09)	35 (7.04)		
Negative	5148 (91.20)	3746 (72.77)	250 (4.86)	720 (13.99)	432 (8.39)		
PM						155.845	< 0.001
Positive	507 (8.98)	275 (54.24)	15 (2.96)	114 (22.49)	103 (20.32)		
Negative	5138 (91.02)	3845 (74.83)	248 (4.83)	681 (13.25)	364 (7.08)		
TB						14.265	0.003
Positive	1675 (29.67)	1186 (70.81)	96 (5.73)	266 (15.88)	127 (7.58)		
Negative	3970 (70.33)	2934 (73.90)	167 (4.21)	529 (13.32)	340 (8.56)		
Cryp						11.276	0.010
Positive	142 (2.52)	96 (67.61)	6 (4.23)	33 (23.24)	7 (4.93)		
Negative	5503 (97.48)	4024 (73.12)	257 (4.67)	762 (13.85)	460 (8.36)		
Candidiasis						33.133	< 0.001
Positive	2717 (48.13)	1911 (70.33)	112 (4.12)	446 (16.42)	248 (9.13)		
Negative	2928 (51.87)	2209 (75.44)	151 (5.16)	349 (11.92)	219 (7.48)		
CMV						20.390	< 0.001
Positive	369 (6.54)	251 (68.02)	7 (1.90)	76 (20.60)	35 (9.49)		
Negative	5276 (93.46)	3869 (73.33)	256 (4.85)	719 (13.63)	432 (8.19)		
PCP						5.411	0.144
Positive	1190 (21.08)	881 (74.03)	46 (3.87)	178 (14.96)	85 (7.14)		
Negative	4455 (78.92)	3239 (72.70)	217 (4.87)	617 (13.85)	382 (8.57)		
EBV						2.865	0.413
Positive	474 (8.40)	350 (73.84)	22 (4.64)	72 (15.19)	30 (6.33)		
Negative	5171 (91.60)	3770 (72.91)	241 (4.66)	723 (13.98)	437 (8.45)		

*HCV* hepatitis C virus, *HBV *hepatitis B virus, *PM *Penicillium marneffei, *TB *tuberculosis, *Cryp *cryptococcus, *CMV *cytomegalovirus, *PCP *pneumocystispneumonia, *EBV *Epstein–Barr virus

### Risk factors for anemia among hospitalized HIV-infected patients

This study used multifactorial logistic regression to identify factors that were independently related to anemia. The final results were presented in Table 3. CD4 counts of < 50 cells/µl (aOR = 2.221, 95%CI = [1.775, 2.779]), CD4 counts of 50–199 cells/µl (aOR = 1.659, 95%CI = [1.327, 2.073]), female (aOR = 1.644, 95%CI = [1.436, 1.881]) co-infected with HCV (aOR = 1.465, 95%CI = [1.071, 2.002]), PM (aOR = 2.356, 95%CI = [1.950, 2.849]), or TB (aOR = 1.198, 95%CI = [1.053, 1.365]) were significantly related to an increased risk of anemia. Age and co-infection with HBV, Cryp, Candidiasis, CMV, PCP, or EBV were not correlated with the prevalence of anemia.

**Table 3:** Multivariate analysis of opportunistic infections associated with anemia

Variables					aOR 95% CI		P-value
	B	S.E	Wald	aOR	Lower limit	Upper limit	
HCV							
Negative	0			1 (ref.)			
Positive	0.382	0.159	5.729	1.465	1.071	2.002	0.017
PM							
Negative	0			1 (ref.)			
Positive	0.857	0.097	78.722	2.356	1.950	2.849	< 0.001
TB							
Negative	0			1 (ref.)			
Positive	0.181	0.066	7.542	1.198	1.053	1.365	0.006
CD4 counts, cells/μl							
> 350	0			1 (ref.)			
200–350	0.231	0.129	3.197	1.260	0.978	1.623	0.074
50–199	0.506	0.114	19.754	1.659	1.327	2.073	< 0.001
< 50	0.798	0.114	48.625	2.221	1.775	2.779	< 0.001
Sex							
Male	0			1 (ref.)			
Female	0.497	0.069	51.968	1.644	1.436	1.881	< 0.001

*HCV* hepatitis C virus, *PM *Penicillium marneffei, *TB *tuberculosis, *OR* odds ratio, *CI* confidence interval

## Discussion

Anemia is a prevalent public health issue worldwide that has a significant impact on health as well as socioeconomic development. The prevalence of anemia in China has been at a high level [16]. In the current study, we examined the incidence and related elements of anemia in hospitalized people living with HIV in Guangxi, China. The results of our study showed the prevalence of anemia in the region to be 27.02%, and most of these patients were moderately anemic. The independent relevant elements for anemia were low CD4 count, females, and co-infection with PM, TB, or HCV.

The total prevalence of anemia in Guangxi, China was similar to previous studies in Ethiopia (23.1%) [17], Poland (25.2%) [18], and the USA (female: 24%, male: 28%) [19]. The prevalence of anemia in the present study was lower compared to previous studies reported in Xinjiang, China (38.9%) [14] and Fujian, China (55.15%) [12]. The possible causes are differences in demographics or different stages of HIV infection. However, in the current study, the prevalence of anemia among people living with HIV was greater than that previously reported in Beijing, China (9.8%) [11]. This difference could be explained by the higher proportion of urban patients included and managed in a hospital-based setting [20], which is more likely to diagnose early anemia and take timely and effective action. Meanwhile, compared to rural subjects, urban subjects were likely to have obtained sufficient nutrition and better healthcare. In addition, most of the participants in the present study were in a state of deteriorating immune function, with 71.57% of patients having CD4 cell counts of < 200 cells/µL. The present study supports previous studies suggesting that anemia is prevalent in HIV-infected patients, with most patients in the moderate anemia stage.

There are relatively few studies on PM because it is endemic only in Southeast Asia and Southern China. Our findings indicated that the prevalence of anemia among HIV-infected patients co-infected with PM was 45.76%. Another retrospective study from Fujian Province, China explored 26 patients with PM infection hospitalized from September 2005 to April 2014 [21]. The study was conducted to evaluate treatment and outcomes by comparing clinical data and laboratory parameters in people living with HIV and people living without HIV with PM infection. A total of 74% of patients with HIV co-infected with PM were anemic. Nevertheless, the included sample of the study was small which focused primarily on clinical symptoms in patients with PM infection and did not exclude potential confounding factors for anemia. Results showed that PM infection was a powerful individual factor associated with anemia in HIV-infected patients.

Anemia was more commonly seen in HIV-infected individuals with TB than in HIV-infected individuals without TB. Similar to the current findings, a study from southern India [22] showed a strong independent association between TB and anemia. Patients with TB were 1.6-fold more likely to be anemic compared to those who did not have TB. Chronic diseases such as TB are popular causes of anemia in HIV-infected patients [23]. Furthermore, causes of TB-associated anemia include chronically infected [24] and nutritionally deficient [25] patients, thereby resulting in suppressed erythropoiesis. However, the etiology of TB-associated anemia in Chinese HIV-infected patients is not well understood as relevant laboratory examinations of red blood cells were not included in this study.

HIV and HCV co-infection is very common because of their similar transmission routes. HIV infection increases HCV viral load and may accelerate HCV disease progression [26]. In the meantime, ribavirin (RBV), a drug frequently used to treat HCV infection, is known to induce hemolytic anemia [27]. Maria Buti et al. reported that side effects of HIV co-infected with HCV during drug treatment include anemia, leukopenia, and depression [28]. In our research, we found that HCV infection was one of the related factors for anemia and this was consistent with earlier studies. Nevertheless, the precise mechanisms causing anemia in patients co-infected with HIV and HCV remain to be further investigated.

Previous studies have reported that the causes of anemia in HIV-infected patients were diverse [6, 7, 29–31]. The current research findings indicated that a lower CD4 count was an independent correlate of anemia in HIV-infected patients. Our findings were consistent with studies reported in Uganda [31], USA [32], and Spain [8]. In addition, the prevalence and severity of anemia increasing with a decrease in CD4 count was discovered. The relationship between CD4 counts and anemia is likely because of increased viral burden as the disease progresses, which may contribute to anemia through increased cytokine-mediated bone marrow suppression [6, 33]. Hence, we identified that the prevalence of OIs decreases as CD4 counts increase. Decreased CD4 counts indicate a loss of immunity in HIV-infected patients and that they may be more susceptible to OIs [34]. Meanwhile, some of the OIs such as TB and PM may infiltrate the bone marrow to inhibit red blood cell production [35].

Current research showed that female patients were more prone to anemia than males. Findings were consistent with those previously published by Harding et al. [36] and Ferede et al. [37] Women's higher prevalence of anemia might be caused by menstrual blood loss, intrauterine devices (IUD) use, and so on [38]. Meanwhile, the high prevalence of anemia in women is associated with malnutrition and nutritional deficiencies, including deficiencies of vitamin B12, folic acid, and iron [39, 40], which directly contribute to anemia.

In the present study, no association was found between anemia and age. However, Shen et al. [13] from China reported that increasing age was remarkably correlated with an increased risk of anemia, but did not correlate with gender. In contrast, Dai et al. found women and older age to be risk factors for anemia [11]. However, the present study did not find a meaningful association between age and anemia in HIV-infected patients through logistic regression models. The main reason for the difference in findings may be the diverse age and gender distribution of the study samples. In addition, the geographical environment, economic conditions, medical policies, and dietary habits of the region under investigation can also affect the results of the study.

In conclusion, this study particularly assessed the relationship between anemia and OIs among hospitalized people living with HIV in Guangxi, China. The present research indicated that HCV, PM, TB, and being female were independently related elements for anemia in people living with HIV. Results confirmed and emphasized that OIs were independently related elements for anemia among HIV patients.

For the first time, the present study reported the correlation between anemia and OIs in HIV-infected patients in Guangxi, China. It also provided a statistical description of anemia and OIs in HIV-infected patients. The current study included a large sample size and was a good representation. Nevertheless, there are still some limitations of this study. First, most of the study population was sourced from Guangxi, China, and did not represent the full population of people living with HIV in China. Second, data were obtained from the electronic medical records of the hospital information system. This prevents us from elucidating the association between anemia and time-varying variables, therefore, the effect of treatment factors on anemia could not be observed. Third, the choice of a retrospective study approach in this study limited the explanation of the causality between anemia and associated factors in people living with HIV. Therefore, exploring the mechanisms of anemia in people living with HIV should be the next step.

## Conclusions

The present research revealed that anemia was prevalent among hospitalized people living with HIV in Southeastern China. Most patients with anemia were in the mild to moderate stage. Lower CD4 counts, being female, and being co-infected with PM, HCV, or TB were independent correlates of anemia in hospitalized people living with HIV. Therefore, people living with HIV should be regularly screened for anemia to improve treatment outcomes and healthcare providers should select appropriate ART plans. The discoveries will greatly help clinicians to better prevent and intervene anemia in people living with HIV.

## Data Availability

The datasets generated and/or analyzed during the current study are not publicly available because of ethical and legal reasons but are available from the corresponding author Jiannan Lv on reasonable request.
